# Fragment library screening reveals remarkable similarities between the G protein-coupled receptor histamine H_4_ and the ion channel serotonin 5-HT_3A_

**DOI:** 10.1016/j.bmcl.2011.06.123

**Published:** 2011-09-15

**Authors:** Mark H.P. Verheij, Chris de Graaf, Gerdien E. de Kloe, Saskia Nijmeijer, Henry F. Vischer, Rogier A. Smits, Obbe P. Zuiderveld, Saskia Hulscher, Linda Silvestri, Andrew J. Thompson, Jacqueline E. van Muijlwijk-Koezen, Sarah C.R. Lummis, Rob Leurs, Iwan J.P. de Esch

**Affiliations:** a Leiden/Amsterdam Center of Drug Research (LACDR), Division of Medicinal Chemistry, Faculty of Sciences, VU University Amsterdam, De Boelelaan 1083, 1081 HV Amsterdam, The Netherlands; b Griffin Discoveries BV. De Boelelaan 1083, Room P-246, 1081 HV Amsterdam, The Netherlands; c Department of Biochemistry, University of Cambridge, Tennis Court Road, Cambridge CB2 1QW, UK

**Keywords:** Fragment-based lead discovery (FBLD), Chemogenomics, Serotonin 5-HT_3A_ receptor, Histamine H_4_ receptor, Dual activity ligand, G-protein coupled receptor (GPCR), Ligand gated ion channel (LGIC)

## Abstract

A fragment library was screened against the G protein-coupled histamine H_4_ receptor (H_4_R) and the ligand-gated ion channel serotonin 5-HT_3A_ (5-HT_3A_R). Interestingly, significant overlap was found between H_4_R and 5-HT_3A_R hit sets. The data indicates that dual active H_4_R and 5 HT_3A_R fragments have a higher complexity than the selective compounds which has important implications for chemical genomics approaches. The results of our fragment-based library screening study illustrate similarities in ligand recognition between H_4_R and 5-HT_3A_R and have important consequences for selectivity profiling in ongoing drug discovery efforts on H_4_R and 5-HT_3A_R. The affinity profiles of our fragment screening studies furthermore match the chemical properties of the H_4_R and 5-HT_3A_R binding sites and can be used to define molecular interaction fingerprints to guide the in silico prediction of protein-ligand interactions and structure.

## Graphical abstract

**Figure:**
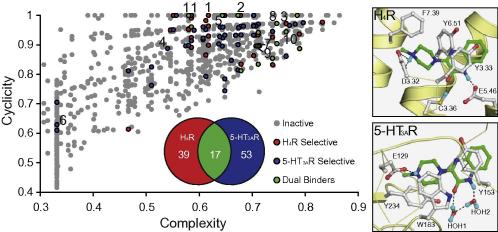

Fragment-based lead discovery (FBLD) uses low molecular weight compounds as starting points for hit and lead optimization. Compared to the drug-like compounds that are screened in typical high-throughput screening campaigns, fragments are better able to cover the corresponding chemical space. Consequently, typical fragment libraries consist of about 1000 small molecules.^1^ Biochemical and biophysical techniques are used to detect the low affinity fragment binding. Ligand efficiency (LE), defined as the binding energy of the ligand (ΔG in kcal mol^−1^) per non-H atom (Heavy Atoms, HA), is used to select the most promising hits and guide the optimization studies.^2^ Typical hit rates for a fragment library screen are considerably higher than for the high throughput screening of drug-like compounds.^3^ The higher complexity of the latter compounds drastically reduces the chances of perfect complementarity with the biological targets. Thus, fragments are particularly suited to probe the binding site of receptors,^4,5^ and are therefore ideal tools in chemogenomic approaches that link chemical with biological space.^6^ In chemogenomics studies the effect of a wide array of chemicals on a wide array of biological targets is investigated.^7^ The resulting two-dimensional matrix of targets versus hit compounds is useful for the discovery of ligands for novel drug targets and to have better control over the selectivity of ligands and/or drugs. Furthermore, the data can lead to a better understanding of ligand-receptor interactions.

We have screened our fragment library against the histamine H_4_ receptor (H_4_R) for which we have ongoing drug discovery programs. H_4_R fragment hits were grown into potent H_4_R ligands and fragment-merging approaches resulted in efficient scaffold hopping towards new chemical series.^8,9^ The H_4_R is considered a very promising target for treating inflammatory and allergic disorders as well in the modulation of pain and pruritis.^10^

Meanwhile, the same fragment library is being screened against a rapidly expanding variety of targets. Here, we describe a remarkable overlap of the fragment hit set of the H_4_R and the 5-HT_3A_R. This ligand-gated ion channel is a drug target for the treatment of irritable bowel syndrome (IBS) and chemotherapy-induced nausea and vomiting (CINV).^11^ Marketed drugs of 5-HT_3A_R include tropisteron (Navoban®) and palonosetron (Aloxi®). The results of our fragment-based library screening indicate similarities in ligand recognition between H_4_R and 5-HT_3A_R and potential selectivity issues when developing H_4_R or 5-HT_3A_R drugs. On the other side, dual activity compounds might also have clinical advantages. Next to the established role of 5HT_3A_R in IBS, recent findings also suggest a role of H_4_R in this disease. It has been found that an increased innate immune activity in the intestinal mucosa and in blood is found in subpopulations of patients with IBS.^12^ Mast cells and monocytes seem to be particularly important and might indicate that the H_4_R is also involved in this ailment.

We screened the biological activity of a diverse set of 1010 fragment-like molecules against H_4_R and 5-HT_3A_R. The compounds in this library obey general fragment library rules^13^: (i) heavy atoms count ⩽ 22; (ii) *c* log *P* <3; (iii) number of H-bond donors ⩽ 3; (iv) number of H-bond acceptors ⩽ 3; (v) number of rotatable bonds ⩽ 5. The fragments furthermore contain at least one ring structure and do not contain reactive functional groups.^14^ The structural diversity of the library was analysed, among others, by means of a scaffold classification analysis (SCA).^15^ In this analysis, fragments are indexed by two parameters, that is, cyclicity and complexity. Cyclicity is the ratio between ring atoms and side chain atoms (thus, if all the atoms of the molecule belong to the ring structure cyclicity equals one). In addition, the complexity was calculated as a descriptor of the size and shape of the scaffold, taking into account the smallest set of smallest rings, the number of heavy atoms, the number of bonds between the heavy atoms, and the sum of heavy atoms atomic number.^15^ Chemical diversity of the fragment library is furthermore confirmed by the fact that only 1.6% of the pair wise comparisons of the ECFP-4 topological fingerprints of the fragments give Tanimoto similarity values higher than 0.26.^16^

For the H_4_R fragment screen a radioligand displacement study was performed at a 10 μM fragment concentration. Hits were assigned when the fragment displaced 50% or more of the radioligand, resulting in 56 hits (hit rate: 6%). Radioligand binding was measured by displacement of [^3^H]histamine using membranes of HEK293 cells transiently expressing the human H_4_R.^17^ For the hit compounds, affinities were determined by subsequent radioligand displacement studies.

For the 5-HT_3A_R we performed a high throughput functional fragment screen^18^ using a fluorescent readout (Flex Station) applying a fluorescent membrane potential dye. With this screening technique we can identify compounds that have affinity for the receptor and in addition classify the hits as agonists, antagonists or inactive. From this fragment screening we identified 70 hits for the 5-HT_3A_ receptor (hit rate: 7%). Fragments were screened at a concentration of 100 μM using stably expressed human 5-HT_3A_R in HEK293 cells. Binding affinities of hits were determined using radioligand binding studies measuring [^3^H]granisetron binding using membranes of HEK293 cells expressing the human 5-HT_3A_R.^18^

The SCA plot^15^ in Figure 1a shows the distribution of 5-HT_3A_R selective hits, H_4_R selective hits, and dual 5-HT_3A_R/H_4_R hits in the chemical space covered by the fragment library and demonstrates the structural diversity of the fragment hits. Interestingly, significant overlap between the H_4_R and 5-HT_3A_R hit sets occur, for example, 24% of the 5-HT_3A_R hits also bind H_4_R and 30% of the H_4_R hits also bind 5-HT_3A_R (Fig. 1b). This is ca. 10% higher than any other overlap between non-related targets that we have screened so far. In Table 1 some selective H_4_R ligands, selective 5-HT_3A_R ligands as well as compounds with affinity for both receptors are displayed. Dual hits **7**, **8**, **11** have comparable affinities for 5-HT_3_R and H_4_R, while dual hit **9** has 500-fold selectivity for 5-HT_3_R over H_4_R, and hit **10** has 200-fold for H_4_R over 5-HT_3_R (Table 1).

Many of the dual H_4_R/5-HT_3A_R ligands contain a quinazoline, quinoxaline, aminopyrimidine, imidazole, or benzimidazole scaffold in combination with a positively ionizable ring system (Table 1). Figure 1c shows that most of these dual H_4_R/5-HT_3A_R fragments have a higher complexity than the H_4_R and 5-HT_3A_R selective fragments. The structural complexity of 71% of the dual 5-HT_3A_/H_4_R fragments is 0.7 or higher, while 79% of the H_4_R selective hits and 74% of the 5-HT_3A_R selective fragments is lower than 0.7. While earlier chemoinformatics analyses suggested that selective ligands are more complex in terms of pharmacophore features^4^ and molecular shape^19^, our fragment-based chemogenomics study suggests a more delicate balance between ligand complexity and target selectivity. Our fragment library screening data indicate that fragments need to have high enough complexity to hit several targets, but low enough complexity to be too specific for a single site. This is in line with the theoretical model by Hann and co-workers^4^ that describes probability of finding a hit when considering the complexity of the ligand. The probability of detecting a binding event is given by multiplying the probability of matching features and the probability of being able to detect low affinity binders. Our experimental data set shows that indeed the chance of finding fragment hits on two different targets favors higher complexity compounds. The relatively high complexity of the overlapping H_4_R and 5-HT_3A_R fragment hit set is furthermore a clear indication that the ligand recognition profiles of these receptors are similar. Figure 2 shows the chemical similarity of the fragment library compared to serotonin and histamine (determined by Pipeline Pilot ECFP-4 circular fingerprint^20^ Tanimoto similarity coefficients (Tc)). While some of the H_4_R selective fragments (38%) are chemically similar to histamine (i.e., ECFP-4 Tc >0.26 including **2** (Table 1)), none of the 5-HT_3A_R selective fragments share chemical similarity serotonin-like, and only two of the dual binders are histamine-like (including **11** see Table 1). These data show that complex H_4_R/5-HT_3A_R dual fragments are dissimilar from the (less complex) endogenous ligands of H_4_R and 5-HT_3A_R.

The higher complexity of the dual H_4_R and 5-HT_3A_R fragments is further illustrated by the analysis of the physical-chemical distributions of fragment hits (Fig. 3). Whereas most properties are similar when comparing the selective and the dual activity fragments (see Table S1, S2 and Figure S1 for details^21^), the number of rings and the heavy atom count (and associated molecular weight) are higher for the dual activity hits compared to for H_4_R and 5-HT_3A_R selective fragments (Fig. 3 and Table S2).

The fragment screening does not only illustrate similarities in H_4_R and 5-HT_3A_R binding profiles, but also identifies subtle differences between the properties of selective receptor ligands. Figure 3 shows that the number of H-bond donor atoms is significantly higher for the H_4_R selective fragments (on average 1.7 H-bond donors) than for 5-HT_3A_R selective fragments (on average 0.8 H-bond donors). This can be correlated with the H_4_R ligand pharmacophore^22^ that contains two H-bond donors. In the H_4_R binding pocket, these features are complementary to two negatively charged residues, D3.32 and E5.46 (Fig. 4).^22^ As a result of these strong non-hydrophobic interactions^23^ between ionizable H-bonding partners, many H_4_R ligands (including the high affinity endogenous ligand histamine) can bind the receptor with a high lipophilic efficiency,^24^ explaining the relatively low *c* log *P* values of H_4_R ligand (Fig. 3). In the 5-HT_3A_R binding pocket one essential glutamate H-bond interaction partner (E129) has been identified (Fig. 4).^25^ Ligand binding to 5-HT_3A_R is furthermore largely determined by aromatic interactions like π–π stacking and cation-π interactions (W183, W195, Y141, Y143, Y153, Y234, see Fig. 4)^25,26^, matching the requirement of a lower number of H-bond donors (and somewhat higher hydrophobicity) for 5-HT_3A_R ligands compared to H_4_R ligands. In line with the notion that the H_4_R binding site contains two essential H-bonding acceptor atoms and the 5-HT_3A_R site only one such atom, is the observation that fragment **10** possesses affinity for both 5-HT_3A_R and H_4_R, whereas the analogous fragment **3** that lacks an NH_2_ group, only shows affinity for the H_4_R.

Figure 4 demonstrates how the affinity profiles from our fragment screening studies correspond with the chemical properties of the H_4_R and 5-HT_3A_R binding sites and can be used to derive molecular interaction fingerprints^27^ and validate structural models of protein-ligand complexes.^28^ In both H_4_R^17^ and 5-HT_3A_R models (see Supplementary data for a description of the protein modeling procedure), the positively ionizable piperazine group of the dual H_4_R/5-HT_3A_R hit **8** forms a salt bridge (D3.32 in H_4_R, E129 in 5-HT_3A_R) and makes cation-π (F7.39 in H_4_R, Y234 and W183 in 5-HT_3A_R) and aromatic π–π stacking interactions (Y3.33 and Y6.51 in H_4_R, Y153 in 5-HT_3A_R). Interestingly, while C3.36 and E5.46 are proposed to act as H-bond donor and acceptor to the carboxamide group of **10** in H_4_R, two water molecules (which form a conserved protein-ligand H-bond interaction network in several crystal structures of the homologous AChBP^29^) fulfill the same role in 5-HT_3A_R. The binding mode modes of **8** presented in Figure 4 do not only match the fragment-based chemogenomics analysis reported in the current study, but are also supported by earlier reported site-directed mutagenesis studies, underlining the important role of E129, W183, Y153, and Y234^25,26^ in ligand binding to 5-HT_3A_R, and the essential role of D3.32 and E5.46 in H_4_R-ligand interactions.^22–24^ The binding orientation of **8** is furthermore in line with previously experimentally validated ligand binding modes in H_4_R.^17^

Chemogenomics analyses of inter-gene family ligand promiscuity is of growing interest.^30^ Although GPCRs and LGICs obviously have a very different protein architecture, their ligand-binding sites can obviously bind similar (sub)structures. In this respect, the special chemical taxonomy of serotonin, that binds to several GPCRs and one ion-channel (5-HT_3A_) has been previously noted.^31^ Moreover, Mestres and co-workers have recently reported a striking cross-pharmacology between aminergic GPCRs and the 5-HT_3_ receptors in their *in silico* target profiling platform.^32^ Our fragment screening studies complement these findings by identifying relatively high fragment cross-reactivity between H_4_R and 5-HT_3A_R (Fig. 1b) and demonstrate that fragments are ideally suited to interrogate ligand binding sites. In the hit optimization phase, selectivity for either H_4_R or 5-HT_3A_R can be achieved, although in some cases this might proof complicated. This is illustrated by recent publications from Abbott Laboratories.^33,34^ These studies, that are part of their H_4_R drug development program, describe the in vitro and in vivo characterization of the H_4_R ligands **12** and **13** (A-940894). Intriguingly, both these compounds (Fig. 5) show strong inhibition at the 5-HT_3A_R receptor (98% inhibition at 10 μM). It is noted that these compounds contain the 2-amino-4-piperazine-pyrimidine scaffold that was also identified as binding to both H_4_R and 5-HT_3A_R in our fragment-screening (fragment 10, Table 1).

In conclusion, the present study identifies a significant overlap between the hit fragment set for H_4_R and 5-HT_3A_R, illustrating similarities in ligand recognition and suggests that fragment-based chemogenomics analysis and molecular modeling building can be used to efficiently navigate chemical space during hit optimization in programs aimed to develop selective leads or compounds with a dual activity profile.

## Figures and Tables

**Figure 1:**
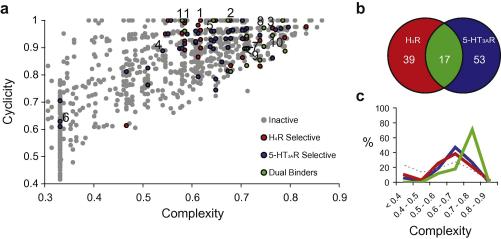
(a) SCA plot showing the hit distribution for the H_4_R (Red), the 5-HT_3A_R (blue), the 5-HT_3A_R and H_4_R (green) as well as inactives, compounds which do not bind H_4_R or 5HT_3A_R (grey). Hits presented in Table 1 are labeled by their corresponding number. (b) Schematic representation of the overlap between H_4_R and 5**-**HT_3A_R ligands. (c) Distribution of the complexity of H_4_R selective fragments (red line), 5-HT_3A_R selective fragments (blue line), dual H_4_R/5-HT_3A_R fragments (green line), and inactives (dotted grey line).

**Figure 2:**
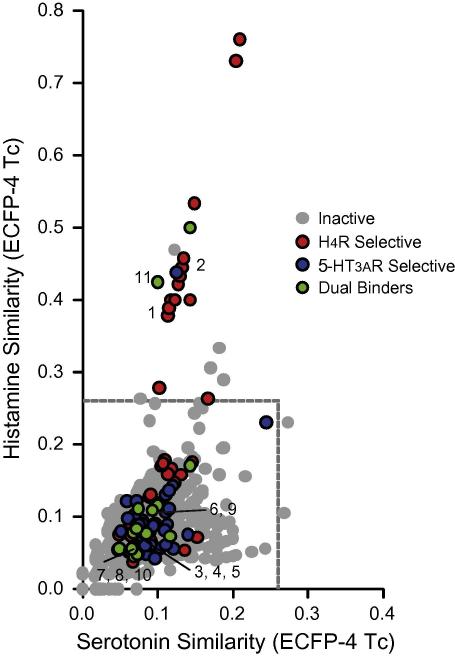
Chemical similarity (ECFP-4 Tc) between dual and selective hits of H_4_R and 5-HT_3A_R and the endogenous ligands of H_4_R (histamine) and 5-HT_3A_R (serotonin).

**Figure 3:**
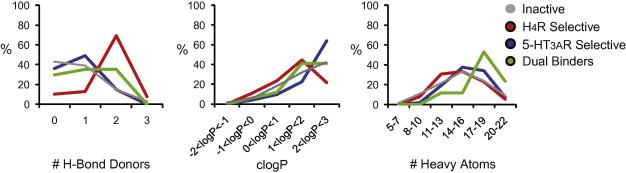
Distribution of physical-chemical properties that discriminate H_4_R selective fragments (red), 5-HT_3A_R selective fragments (blue line), dual H_4_R/5-HT_3A_R fragments (green), and inactives (grey dotted line). Distributions of other physical-chemical properties do not discriminate between the sets as shown in Figure S1

**Figure 4:**
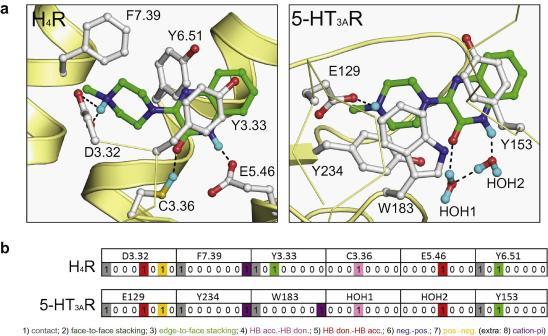
Panel A shows the predicted binding modes of the dual H_4_R/5-HT_3A_R hit **8** (green carbon atoms, see Table 1 for molecular structure) in structural models of H_4_R and 5-HT_3A_R. Parts of the backbone of transmembrane (TM) helices 3, 5, 6 and 7 (the top TM3 is not shown for clarity) in H_4_R and loops A, B, C and E of the extracellular ligand-binding domain (ECD) of 5-HT_3A_R are represented by light yellow ribbons. Important binding residues are depicted as ball-and-sticks with grey carbon atoms. Oxygen, nitrogen, and hydrogen atoms are colored red, blue, and cyan, respectively. H-bonds described in the text are depicted by black dots. The molecular interaction fingerprint (IFP)^27^ bit strings of **8** in H_4_R and 5-HT_3A_R are compared in panel B.

**Figure 5:**
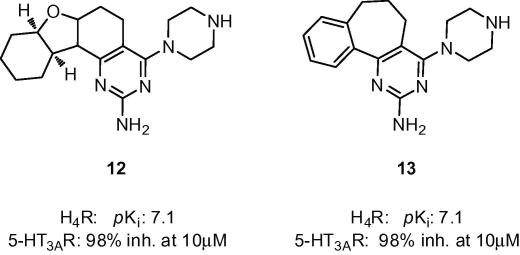
Compounds in preclinical trials by Abbott.

**Table 1:** Structures of fragments that bind solely H_4_R (1–3), solely 5-HT_3A_R (4–6) and both H_4_R and 5-HT_3A_R (7–11)

#	H_4_R/5-HT_3A_R	Structure	Affinity (p*K*_i_)	
			H_4_R^a^	5-HT_3A_R^c^
1			7.0 ± 0.1	n.a.
2			6.2 ± 0.1	n.a.
3			6.7 ± 0.0	n.a.
4			n.a.	6.1 ± 0.2
5			n.a.	6.0 ± 0.0
6			n.a.	6.1 ± 0.1
7			6.2 ± 0.0	6.6 ± 0.3
8			7.2 ± 0.0	7.9 ± 0.3
9			6.1 ± 0.1^b^	8.8 ± 0.1
10			8.2 ± 0.1	5.9 ± 0.1
11			6.2 ± 0.1^b^	5.9 ± 0.3

n.a.: Non active.

a Measured by displacement of [^3^H]histamine binding using membranes of HEK293 cells transiently expressing the human H_4_R. p*K*_i_’s are calculated from at least three independent measurements as the mean ± SEM.

b Determined using membranes of SK-N-MC cells transiently expressing the human H_4_R. p*K*_i_’s are calculated from at least three independent measurements as the mean ± SEM.

c p*K*_i_: Measured by displacement of [^3^H]granisetron binding using membranes of HEK293 cells expressing the human 5-HT_3A_R. p*K*_i_’s are calculated from at least two independent measurements as the mean ± SEM.

## Supplementary data

Supplementary data Distributions of physical-chemical properties of fragment hits, descriptions of H_4_R and 5-HT_3A_R protein modeling procedures and experimental details of H_4_R and 5-HT_3A_R screens.
