# Supplementary material for: Fragment library screening reveals remarkable similarities between the G protein-coupled receptor histamine H4 and the ion channel serotonin 5-HT3A
Source: Bioorg Med Chem Lett. 2011 Sep 15;21(18):5460–4. doi: 10.1016/j.bmcl.2011.06.123 (PMC3235552; doi:10.1016/j.bmcl.2011.06.123)
Supplement: Supplementary data — Distributions of physical-chemical properties of fragment hits, descriptions of H4R and 5-HT3AR protein modeling procedures and experimental details of H4R and 5-HT3AR screens. [file mmc1.doc]

**Supporting Information**

Fragment library screening reveals remarkable similarities between the G protein-coupled receptor histamine H_4_ and the ion channel serotonin 5-HT_3A_

Mark H. P. Verheij ^a^, Chris de Graaf ^a^, Gerdien E. de Kloe ^a^, Saskia Nijmeijer ^a^, Henry F. Vischer ^a^, Rogier A. Smits ^b^, Obbe P. Zuiderveld ^a^, Saskia Hulscher ^a^, Linda Silvestri ^c^, Andrew J. Thompson ^c^, Jacqueline E. van Muijlwijk-Koezen ^a^, Sarah C. R. Lummis ^c^, Rob Leurs ^a^ and Iwan J. P. de Esch ^a*^

*^a^ Leiden/Amsterdam Center of Drug Research (LACDR), Division of Medicinal Chemistry, Faculty of Sciences, VU University Amsterdam, De Boelelaan 1083, 1081 HV Amsterdam, The Netherlands.*

*^b^ Griffin Discoveries BV. De Boelelaan 1083, Room P-246, 1081 HV Amsterdam, The Netherlands*

*^c^ Department of Biochemistry, University of Cambridge, Tennis Court Road, Cambridge CB2 1QW, UK*

**Table of contents:**

Figure S1 Page S2

Table S1 Page S3

Table S2 Page S4

Experimental Section Page S8


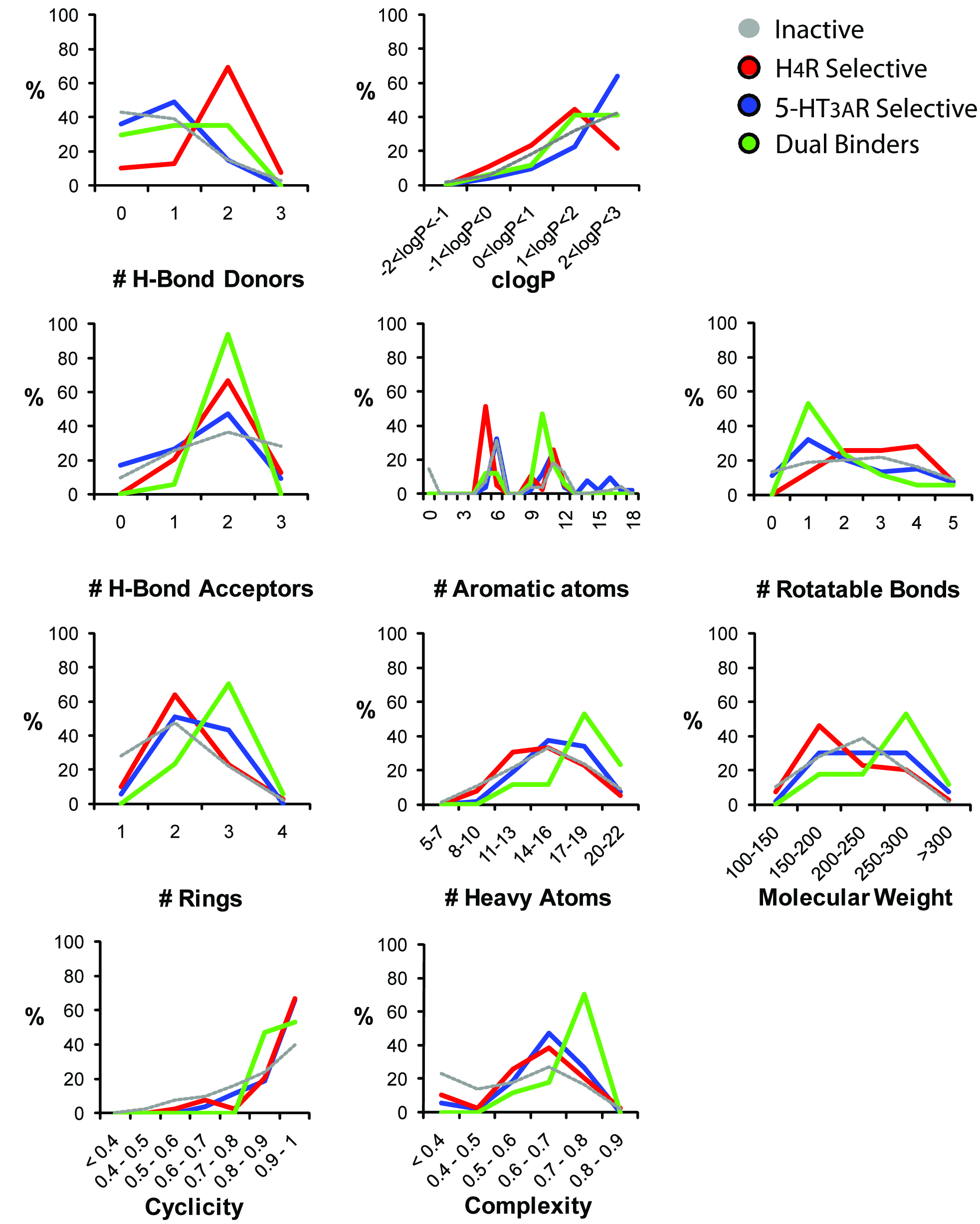


**Figure S1:** Distribution physical-chemical properties that discriminate 5-HT_3A_R selective fragments (blue line), H_4_R selective fragments (red line), dual H_4_R/5-HT_3A_R fragments (green line), and inactive compounds that do not bind H_4_R or 5HT_3A_R (grey dotted line)

**Table S1:** Average numbers with their corresponding standard deviations for the physical-chemical properties.

|  |  | **Compl.** | **Cyclicity** | **ECFP-4 Tc Histamine** | **ECFP-4 Tc Serotonine** | **HBA** | **HBD** | **# heavy atoms** | **# rot. bonds** | **# rings** | **# aromatic atoms** | **clogP** | **MW** |
| --- | --- | --- | --- | --- | --- | --- | --- | --- | --- | --- | --- | --- | --- |
|  |  |  |  |  |  |  |  |  |  |  |  |  |  |
| **H_4_R Selective** | **Mean** | **0.61** | **0.91** | **0.25** | **0.12** | **1.92** | **1.74** | **14.72** | **2.92** | **2.18** | **7.49** | **1.04** | **207.13** |
|  | Sd | 0.13 | 0.12 | 0.19 | 0.04 | 0.58 | 0.75 | 3.12 | 1.18 | 0.64 | 2.85 | 0.85 | 49.18 |
|  |  |  |  |  |  |  |  |  |  |  |  |  |  |
| **5-HT_3A_R Selective** | **Mean** | **0.63** | **0.90** | **0.09** | **0.09** | **1.49** | **0.79** | **15.81** | **2.11** | **2.38** | **10.08** | **1.99** | **219.59** |
|  | Sd | 0.10 | 0.09 | 0.06 | 0.03 | 0.89 | 0.69 | 2.57 | 1.49 | 0.60 | 3.74 | 0.85 | 40.98 |
|  |  |  |  |  |  |  |  |  |  |  |  |  |  |
| **H_4_R and 5-HT_3A_R** | **Mean** | **0.71** | **0.92** | **0.12** | **0.09** | **1.94** | **1.06** | **17.76** | **1.88** | **2.82** | **9.18** | **1.66** | **252.46** |
|  | Sd | 0.06 | 0.06 | 0.13 | 0.03 | 0.24 | 0.83 | 2.75 | 1.22 | 0.53 | 2.21 | 0.82 | 45.51 |
|  |  |  |  |  |  |  |  |  |  |  |  |  |  |
| **Inactive** | **Mean** | **0.55** | **0.82** | **0.08** | **0.08** | **1.83** | **0.78** | **14.87** | **2.37** | **1.99** | **7.81** | **1.62** | **210.29** |
|  | Sd | 0.15 | 0.14 | 0.04 | 0.04 | 0.95 | 0.80 | 3.44 | 1.51 | 0.77 | 4.55 | 1.00 | 47.36 |

**Table S2:** Physchem properties for the different hit sets. Left side: Absolute numbers; Right side: Percentage (%).

|  |  | **Complexity** |  |  |  |  |  | **Complexity** |  |  |
| --- | --- | --- | --- | --- | --- | --- | --- | --- | --- | --- |
|  | H_4_R selective | 5-HT_3A_R selective | H_4_R and 5-HT_3A_R | Inactive |  |  | H_4_R selective | 5-HT_3A_R selective | H_4_R and 5-HT_3A_R | Inactive |
| < 0.4 | 4 | 3 | 0 | 206 |  | < 0.4 | 10 | 6 | 0 | 23 |
| 0.4 - 0.5 | 1 | 1 | 0 | 123 |  | 0.4 - 0.5 | 3 | 2 | 0 | 14 |
| 0.5 - 0.6 | 10 | 10 | 2 | 162 |  | 0.5 - 0.6 | 26 | 19 | 12 | 18 |
| 0.6 - 0.7 | 15 | 25 | 3 | 245 |  | 0.6 - 0.7 | 38 | 47 | 18 | 27 |
| 0.7 - 0.8 | 8 | 14 | 12 | 148 |  | 0.7 - 0.8 | 21 | 26 | 71 | 16 |
| 0.8 - 0.9 | 1 | 0 | 0 | 17 |  | 0.8 - 0.9 | 3 | 0 | 0 | 2 |
| 0.9 - 1 | 0 | 0 | 0 | 0 |  | 0.9 - 1 | 0 | 0 | 0 | 0 |

|  |  | **Cyclicity** |  |  |  |  |  | **Cyclicity** |  |  |
| --- | --- | --- | --- | --- | --- | --- | --- | --- | --- | --- |
|  | H_4_R selective | 5-HT_3A_R selective | H_4_R and 5-HT_3A_R | Inactive |  |  | H_4_R selective | 5-HT_3A_R selective | H_4_R and 5-HT_3A_R | Inactive |
| < 0.4 | 0 | 0 | 0 | 1 |  | < 0.4 | 0 | 0 | 0 | 0 |
| 0.4 - 0.5 | 0 | 0 | 0 | 22 |  | 0.4 - 0.5 | 0 | 0 | 0 | 2 |
| 0.5 - 0.6 | 1 | 0 | 0 | 69 |  | 0.5 - 0.6 | 3 | 0 | 0 | 8 |
| 0.6 - 0.7 | 3 | 2 | 0 | 89 |  | 0.6 - 0.7 | 8 | 4 | 0 | 10 |
| 0.7 - 0.8 | 1 | 6 | 0 | 144 |  | 0.7 - 0.8 | 3 | 11 | 0 | 16 |
| 0.8 - 0.9 | 8 | 10 | 8 | 217 |  | 0.8 - 0.9 | 21 | 19 | 47 | 24 |
| 0.9 - 1 | 26 | 35 | 9 | 359 |  | 0.9 - 1 | 67 | 66 | 53 | 40 |

**Table S2:** Physchem properties for the different hit sets. Left side: Absolute numbers; Right side: Percentage (%).

|  |  | **HBA** |  |  |  |  |  | **HBA** |  |  |
| --- | --- | --- | --- | --- | --- | --- | --- | --- | --- | --- |
|  | H_4_R selective | 5-HT_3A_R selective | H_4_R and 5-HT_3A_R | Inactive |  |  | H_4_R selective | 5-HT_3A_R selective | H_4_R and 5-HT_3A_R | Inactive |
| 0 | 0 | 9 | 0 | 86 |  | 0 | 0 | 17 | 0 | 10 |
| 1 | 8 | 14 | 1 | 233 |  | 1 | 21 | 26 | 6 | 26 |
| 2 | 26 | 25 | 16 | 329 |  | 2 | 67 | 47 | 94 | 37 |
| 3 | 5 | 5 | 0 | 253 |  | 3 | 13 | 9 | 0 | 28 |

|  |  | **HBD** |  |  |  |  |  | **HBD** |  |  |
| --- | --- | --- | --- | --- | --- | --- | --- | --- | --- | --- |
|  | H_4_R selective | 5-HT_3A_R selective | H_4_R and 5-HT_3A_R | Inactive |  |  | H_4_R selective | 5-HT_3A_R selective | H_4_R and 5-HT_3A_R | Inactive |
| 0 | 4 | 19 | 5 | 387 |  | 0 | 10 | 36 | 29 | 43 |
| 1 | 5 | 26 | 6 | 354 |  | 1 | 13 | 49 | 35 | 39 |
| 2 | 27 | 8 | 6 | 134 |  | 2 | 69 | 15 | 35 | 15 |
| 3 | 3 | 0 | 0 | 26 |  | 3 | 8 | 0 | 0 | 3 |

|  |  | **# Heavy Atoms** |  |  |  |  |  | **# Heavy Atoms** |  |  |
| --- | --- | --- | --- | --- | --- | --- | --- | --- | --- | --- |
|  | H_4_R selective | 5-HT_3A_R selective | H_4_R and 5-HT_3A_R | Inactive |  |  | H_4_R selective | 5-HT_3A_R selective | H_4_R and 5-HT_3A_R | Inactive |
| 5-7 | 0 | 0 | 0 | 12 |  | 5-7 | 0 | 0 | 0 | 1 |
| 8-10 | 3 | 1 | 0 | 99 |  | 8-10 | 8 | 2 | 0 | 11 |
| 11-13 | 12 | 10 | 2 | 193 |  | 11-13 | 31 | 19 | 12 | 21 |
| 14-16 | 13 | 20 | 2 | 300 |  | 14-16 | 33 | 38 | 12 | 33 |
| 17-19 | 9 | 18 | 9 | 214 |  | 17-19 | 23 | 34 | 53 | 24 |
| 20-22 | 2 | 4 | 4 | 83 |  | 20-22 | 5 | 8 | 24 | 9 |

**Table S2:** Physchem properties for the different hit sets. Left side: Absolute numbers; Right side: Percentage (%).

|  |  | **# Rings** |  |  |  |  |  | **# Rings** |  |  |
| --- | --- | --- | --- | --- | --- | --- | --- | --- | --- | --- |
|  | H_4_R selective | 5-HT_3A_R selective | H_4_R and 5-HT_3A_R | Inactive |  |  | H_4_R selective | 5-HT_3A_R selective | H_4_R and 5-HT_3A_R | Inactive |
| 1 | 4 | 3 | 0 | 252 |  | 1 | 10 | 6 | 0 | 28 |
| 2 | 25 | 27 | 4 | 428 |  | 2 | 64 | 51 | 24 | 48 |
| 3 | 9 | 23 | 12 | 201 |  | 3 | 23 | 43 | 71 | 22 |
| 4 | 1 | 0 | 1 | 20 |  | 4 | 3 | 0 | 6 | 2 |

|  |  | **# Aromatic Atoms** |  |  |  |  |  | **# Aromatic Atoms** |  |  |
| --- | --- | --- | --- | --- | --- | --- | --- | --- | --- | --- |
|  | H_4_R selective | 5-HT_3A_R selective | H_4_R and 5-HT_3A_R | Inactive |  |  | H_4_R selective | 5-HT_3A_R selective | H_4_R and 5-HT_3A_R | Inactive |
| 0 | 0 | 0 | 0 | 132 |  | 0 | 0 | 0 | 0 | 15 |
| 1 | 0 | 0 | 0 | 0 |  | 1 | 0 | 0 | 0 | 0 |
| 2 | 0 | 0 | 0 | 0 |  | 2 | 0 | 0 | 0 | 0 |
| 3 | 0 | 0 | 0 | 0 |  | 3 | 0 | 0 | 0 | 0 |
| 4 | 0 | 0 | 0 | 0 |  | 4 | 0 | 0 | 0 | 0 |
| 5 | 20 | 2 | 2 | 66 |  | 5 | 51 | 4 | 12 | 7 |
| 6 | 2 | 17 | 2 | 282 |  | 6 | 5 | 32 | 12 | 31 |
| 7 | 0 | 0 | 0 | 0 |  | 7 | 0 | 0 | 0 | 0 |
| 8 | 0 | 0 | 0 | 2 |  | 8 | 0 | 0 | 0 | 0 |
| 9 | 4 | 1 | 1 | 33 |  | 9 | 10 | 2 | 6 | 4 |
| 10 | 1 | 6 | 8 | 35 |  | 10 | 3 | 11 | 47 | 4 |
| 11 | 10 | 13 | 3 | 168 |  | 11 | 26 | 25 | 18 | 19 |
| 12 | 2 | 2 | 1 | 111 |  | 12 | 5 | 4 | 6 | 12 |
| 13 | 0 | 0 | 0 | 0 |  | 13 | 0 | 0 | 0 | 0 |
| 14 | 0 | 4 | 0 | 4 |  | 14 | 0 | 8 | 0 | 0 |
| 15 | 0 | 1 | 0 | 10 |  | 15 | 0 | 2 | 0 | 1 |
| 16 | 0 | 5 | 0 | 19 |  | 16 | 0 | 9 | 0 | 2 |
| 17 | 0 | 1 | 0 | 39 |  | 17 | 0 | 2 | 0 | 4 |
| 18 | 0 | 1 | 0 | 0 |  | 18 | 0 | 2 | 0 | 0 |

**Table S2:** Physchem properties for the different hit sets. Left side: Absolute numbers; Right side: Percentage (%).

|  |  | **# Rotatable Bonds** |  |  |  |  |  | **# Rotatable Bonds** |  |  |
| --- | --- | --- | --- | --- | --- | --- | --- | --- | --- | --- |
|  | H_4_R selective | 5-HT_3A_R selective | H_4_R and 5-HT_3A_R | Inactive |  |  | H_4_R selective | 5-HT_3A_R selective | H_4_R and 5-HT_3A_R | Inactive |
| 0 | 0 | 6 | 0 | 119 |  | 0 | 0 | 11 | 0 | 13 |
| 1 | 5 | 17 | 9 | 170 |  | 1 | 13 | 32 | 53 | 19 |
| 2 | 10 | 11 | 4 | 183 |  | 2 | 26 | 21 | 24 | 20 |
| 3 | 10 | 7 | 2 | 198 |  | 3 | 26 | 13 | 12 | 22 |
| 4 | 11 | 8 | 1 | 148 |  | 4 | 28 | 15 | 6 | 16 |
| 5 | 3 | 4 | 1 | 88 |  | 5 | 7 | 8 | 5 | 10 |

|  |  | **clogP** |  |  |  |  |  | **clogP** |  |  |
| --- | --- | --- | --- | --- | --- | --- | --- | --- | --- | --- |
|  | H_4_R selective | 5-HT_3A_R selective | H_4_R and 5-HT_3A_R | Inactive |  |  | H_4_R selective | 5-HT_3A_R selective | H_4_R and 5-HT_3A_R | Inactive |
| -2<logP<-1 | 0 | 0 | 0 | 16 |  | -2<logP<-1 | 0 | 0 | 0 | 2 |
| -1<logP<0 | 5 | 2 | 1 | 51 |  | -1<logP<0 | 13 | 4 | 6 | 6 |
| 0<logP<1 | 11 | 5 | 2 | 162 |  | 0<logP<1 | 28 | 9 | 12 | 18 |
| 1<logP<2 | 18 | 12 | 7 | 289 |  | 1<logP<2 | 46 | 23 | 41 | 32 |
| 2<logP<3 | 5 | 34 | 7 | 383 |  | 2<logP<3 | 13 | 64 | 41 | 43 |

|  |  | **Molecular Weight** |  |  |  |  |  | **Molecular Weight** |  |  |
| --- | --- | --- | --- | --- | --- | --- | --- | --- | --- | --- |
|  | H_4_R selective | 5-HT_3A_R selective | H_4_R and 5-HT_3A_R | Inactive |  |  | H_4_R selective | 5-HT_3A_R selective | H_4_R and 5-HT_3A_R | Inactive |
| 50-100 | 0 | 0 | 0 | 4 |  | 50-100 | 0 | 0 | 0 | 0 |
| 100-150 | 3 | 1 | 0 | 96 |  | 100-150 | 8 | 2 | 0 | 11 |
| 150-200 | 18 | 16 | 3 | 252 |  | 150-200 | 46 | 30 | 18 | 28 |
| 200-250 | 9 | 16 | 3 | 350 |  | 200-250 | 23 | 30 | 18 | 39 |
| 250-300 | 8 | 16 | 9 | 184 |  | 250-300 | 21 | 30 | 53 | 20 |
| >300 | 1 | 4 | 2 | 15 |  | >300 | 3 | 8 | 12 | 2 |

**Experimental Section:**

**Chemogenomics analyses**

The fragment structures were retrieved from our in-house database as 2D SD format and processed with the Molecular Operating Environment (MOE) software.^1^ The fragments were protonated (standard protonation states at pH=7); physico-chemical properties were calculated using MOE 2D descriptors. The scaffold diversity analysis was performed in MOE, using the freely available sca.svl script.^2^ Fingerprint similarity searches of two databases were performed using the Scitegic Pipeline Pilot.^3^

**5-HT_3A_R assays**

5-HT_3A_R were expressed in HEK293 cells transfected with the human 5-HT_3A_ subunit (PDB ID: P46098) in pcDNA3.1 (Invitrogen Ltd., Paisley, UK). Stably expressing cells were clonally selected with the addition of the G-418 (Sigma Aldrich Company ltd., Dorset, UK). Cells were routinely grown in 90 mm culture dishes containing Dubelco’s Modified Eagle’s Medium DMEM / Glutamax (Gibco, Invitrogen Ltd.) and passaged when 90% confluency was reached.

For fluorescence measurements cells were transferred from 90 mm culture dishes (to maximise fluorescence cells were used when 100% confluent) to black 96 well plates (Greiner Bio-One Ltd., Stonehouse, UK) treated with 0.01% Cultrex Poly-L-Lysine (Trevigen Inc., MD, USA). After 24 hrs, growth medium was removed and the cells incubated for 60 min with 100 µl fluorescent membrane potential dye (FLIPR Membrane Potential Assay Kit, Molecular Devices, Wokingham, UK) dissolved in buffer (mM: 115 NaCl, 1 KCl, 1 MgCl2, 1 CaCl2, 10 HEPES, 10 D-Glucose, pH 7.4). Dye-loaded cells were transferred to a FlexStation II (Molecular Devices) and test compounds added to the cells without removing the dye buffer. Fluorescence was recorded every 2 s for 20 s, to provide a baseline level, after which the compounds were added, and the change in fluorescence recorded every 2 s for a further 80 s. Fluorescence levels were measured using Softmax Pro v4.3 (Molecular Devices) and exported to Microsoft Excel 2002 SP3 for analysis. Compounds were categorised as hits according to the methods described in Thompson et al., 2010.^4^

$B_{L}=B_{\min}+\frac{B_{\max}-B_{\min}}{1+10^{n_{H}\left(\log L_{50}-\log L\right)}}$For radioligand binding HEK293 cells at 90% confluency were scraped into 1 ml of ice-cold HEPES buffer (10 mM, pH 7.4) and frozen. After thawing, they were washed with HEPES buffer and homogenised using a fine-bore syringe. 50 µl of cell membranes were incubated in 0.5 ml HEPES buffer containing 0.7 nM [^3^H]granisetron (~ K_d_) and differing concentrations of the test compound. Competition binding (8 point) was performed on at least three separate plates of transfected cells. Non-specific binding was determined using 1 mM quipazine. Reactions were incubated for at least 24 h at 4°C, to allow compounds with slow kinetics to equilibrate. Incubations were terminated by vacuum filtration using a Brandel cell harvester (Alpha Biotech Ltd., London, UK) onto GF/B filters pre-soaked in 0.3 % polyethyleneimine. Radioactivity was determined by scintillation counting using a Beckman BCLS6500 (Fullerton, California, USA). Data were fit according to the equation:

where L is the concentration of ligand present; BL is the binding in the presence of ligand concentration L; Bmin is the binding when L = 0; Bmax is the binding when L=∞, L50 is the concentration of L which gives a binding equal to (Bmax + Bmin)/2; and n_H_ is the Hill coefficient. K_i_ values were estimated from IC_50_ values using the Cheng-Prusoff equation^5^

$$K_{\text{i}}=\frac{IC_{50}}{1+[L]/K_{\text{d}}}$$

where K_i_ is the equilibrium dissociation constant for binding of the unlabeled antagonist, IC_50_ is the concentration of antagonist that blocks half the specific binding, [L] is the free concentration of radioligand and K_d_ is the equilibrium dissociation constant of the radioligand.

**H_4_R assays**

The H_4_R radioligand displacement screen was performed on homogenized HEK293T cells transiently expressing the human H_4_R. To this end, cells were transfected with 1.25 μg H4R plasmid per million cells using the 25-kDa linear polyethyleneimine. Two days after transfection, cells were collected, washed in PBS, and stored as pellets at -20ºC. Pellets were dissolved in binding buffer and homogenized using a teflon/glass followed by sonification for 3s. Cell homogenates were incubated in 96 well plates with 10μM (final) fragment and 10nM [^3^H]-histamine (13.4 Ci/mmol, Perkin Elmer) and for 1.5 hours at room temperature on a table shaker (750rpm). Incubations were terminated by rapid filtration through polyethyleneimene-coated GF/C (Perkin Elmer) filter plates. Bound radioactivity was quantified in a Wallac microbeta counter (Perkin Elmer).

**Three-dimensional protein modeling and ligand binding mode analysis H_4_R and 5-HT_3A_R**

The H_4_R receptor model was constructed as previously described.^6^ Compound **8** was docked into the H_4_R receptor model using using 20 independent PLANTS^7^ docking runs. The docking pose donating a H-bond to the essential D3.32 residue^8^ with the highest PLANTS score was selected using a protein-ligand interaction fingerprint (IFP) scoring protocol described previously.^9, 10^ The docked H_4_R-ligand complex was then subjected to energy minimization with AMBER^11^ using the AMBER03 force field^12^ by 1,000 steps of steepest descent followed by conjugate gradient until the rms gradient of the potential energy was lower than 0.05 kcal/mol/Å. A twin cut-off (12.0, 15.0 Å) was used to calculate non-bonded electrostatic interactions and the non-bonded pair-list was updated every 25 steps. Force-field parameters of compound **8** were derived using the Antechamber program^11^ and partial charges for the ligands were computed using the AM1-BCC procedure in Antechamber. The Ballesteros-Weinstein residue numbering scheme^13^ was used throughout this manuscript for H_4_R.

A preliminary 5-HT_3A_R model was constructed using MOE^14^ based on the tropisetron-bound AChBP crystal structure (PDB code: 2WNC)^15^ and the human 5-HT3A gene (Q7MZK7). The amino acid sequence alignment used for constructing the receptor model is shown in Figure S2. Structural waters located in the binding pocket of the 2WNC crystal structure which form a conserved protein-ligand H-bond interaction network in several crystal structures of the homologous AChBP^15^ were included in the 5-HT_3A_R model. Compound **8** was docked into this 5-HT_3A_R model using 20 independent PLANTS docking runs. The docking pose donating a H-bond to the essential E129^16^ residue with the highest PLANTS score was selected using the same IFP scoring protocol used for H_4_R and subjected to energy minimization with AMBER using the minimization protocol as described for H_4_R.


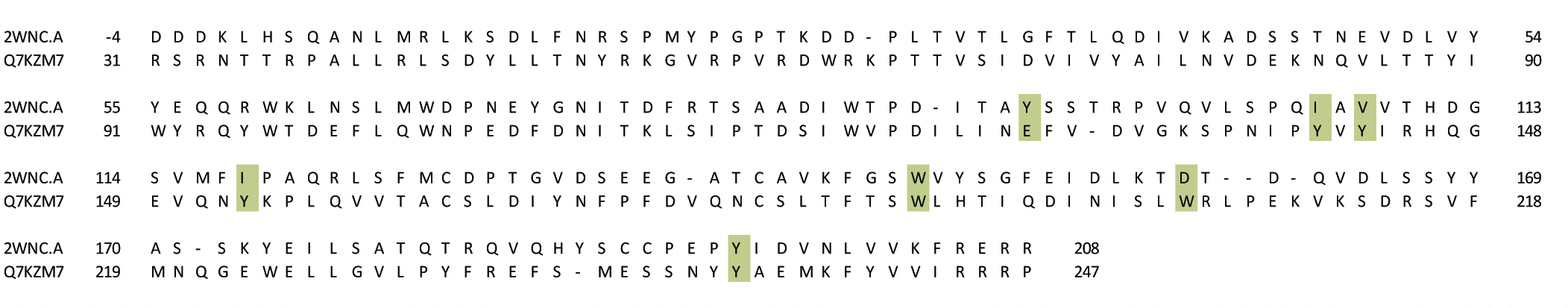


**Figure S2:** Sequence alignment for *Ac-*AChBP (2WNC.A) and 5-HT_3A_R (Q7KZM7). 5-HT_3A_R residues referred to in the paper and their corresponding residues in *Ac*-AChBP are highlighted.

**References:**
